# Suicide risk in patient with diabetes varies by the duration of diabetes: The Korea National Health and Nutrition Examination Survey

**DOI:** 10.1192/j.eurpsy.2024.551

**Published:** 2024-08-27

**Authors:** Y.-C. Kim, Y.-H. Um, H.-J. Seo, J.-H. Jeong

**Affiliations:** Psychiatry, St. Vincent’s Hospital, The Catholic.ac.kr, Suwon, Korea, Republic Of

## Abstract

**Introduction:**

Suicide has a complex relationship with several factors, and it is known that identifying high-risk groups of suicide and managing crisis in advance can help prevent suicide. Moreover in a previous study, it showed that people with chronic diseases often suffer from psychological difficulties such as depression and anxiety, which can influence one to commit suicide. Based on many studies about the relationship between diabetes and depression, 10% of diabetic patients experience major depression, and diabetic patients experience twice as much depression as the general population. But, there are few studies examining the relationship between diabetes and suicide risk, and most of them were targeted for type 1 diabetes only.

**Objectives:**

The objectives of this study were to investigate the suicide risk in diabetic patients, and evaluate the suicide risk varies by the duration of diabetes, using a large population sample in South Korea

**Methods:**

Using the 2019 Korea National Health and Nutrition Examination Survey data, 6,296 adults (aged 19 years or older) were included. Suicidal ideation, suicidal plan, and suicidal behavior of diabetic patients were compared with the general population. After classifying the patients into ≤ 1 year, 2 to 9 years, and 10 years ≤ for the duration of diabetes, we evaluated the relationship between the duration of diabetes and the risk of suicide.

**Results:**

Diabetic patients had higher prevalence of suicidal ideation (9.1%, P<0.001) and suicidal plan (3.6%, P<0.001) than general population. After adjusting for potential confounding factors, suicidal plan (aOR = 3.011, 95% CI = 1.392-6.512) was significantly associated with diabetes. In the 2 to 9 year group of diabetes, we found an increase in risk of suicidal ideation (aOR=2.068, 95% CI=1.219-3.510), suicidal plan (aOR=3.640, 95% CI=1.592-8.320), and suicidal behavior (aOR=6.222, 95% CI=1.759-22.008) after adjusting covariates. However, increase in suicide risk was not observed in the ≤1 year and 10 years ≤ groups after diagnosis of diabetes.

**Conclusions:**

In adults, diabetes is associated with increase in suicide risk. Suicide risk in diabetic patients shows an ‘inverted U-shaped’ depending on the duration of diabetes.

**Disclosure of Interest:**

None Declared

